# Biodegradation of Phenol by *Rhodococcus* sp. Strain SKC: Characterization and Kinetics Study

**DOI:** 10.3390/molecules25163665

**Published:** 2020-08-12

**Authors:** Yujuan Wen, Chaofan Li, Xiaoming Song, Yuesuo Yang

**Affiliations:** 1 Key Laboratory of Regional Environment and Eco-restoration, Ministry of Education, Shenyang University, Shenyang 110044, China; lichaofan1211@126.com (C.L.); songxm@syu.edu.cn (X.S.); yangyuesuo@jlu.edu.cn (Y.Y.); 2 College of New Energy and Environment, Jilin University, Changchun 130021, China

**Keywords:** phenol, *Rhodococcus* sp., kinetic model, biokinetic constants

## Abstract

This study focuses on the kinetics of a pure strain of bacterium *Rhodococcus* sp. SKC, isolated from phenol-contaminated soil, for the biodegradation of phenol as its sole carbon and energy source in aqueous medium. The kinetics of phenol biodegradation including the lag phase, the maximum phenol degradation rate, maximum growth rate (*R_m_*) and maximum yield coefficient (Y) for each *S*_i_ (initial phenol concentration, mg/L) were fitted using the Gompertz and Haldane models of substrate inhibition (R^2^ > 0.9904, RMSE < 0.00925). The values of these parameters at optimum conditions were *μ*_max_ = 0.30 h^−1^, *K_s_* = 36.40 mg/L, and *K*_i_ = 418.79 mg/L, and that means the inhibition concentration of phenol was 418.79 mg/L. By comparing with other strains of bacteria, *Rhodococcus* sp. SKC exhibited a high yield factor and tolerance towards phenol. This study demonstrates the potential application of *Rhodococcus* sp. SKC for the bioremediation of phenol contaminate.

## 1. Introduction

Phenol is a protoplasmic toxin that is common in industrial waste water, originating from coking plants, dyes, varnishes, pharmaceuticals, and pesticides [1]. Due to its high toxicity to life and overall chemical stability, phenol frequently causes the breakdown of wastewater treatment plants [2,3]. Thus, phenol has been listed as one of 126 priority pollutants by the US Environmental Protection Agency [4]; the maximum phenol content in drinking water should be below 1.0 μg/L [5].

Phenol is a member of the phenolic compounds with the chemical formula C_6_H_6_O. It is an important raw material that is needed to produce resins, fungicides, and preservatives, as well as common medicines such as aspirin [6]. This compound is extremely toxic, easily absorbed through the skin, and causes liver and/or kidney damage. At present, phenol has been shown to cause the contamination of the aquatic and atmospheric environment. Moreover, accidental spills of phenol were the main reason for aquatic contamination [7]. From the literature, the concentration of phenol in Isebo River (Nigeria) reached 2110.0 μg/L. As for the sea, the concentration of volatile phenols (including phenol and cresols) in Maoming onshore fishery (China) reached 26 μg/L. In addition, concentrations of phenol in the atmosphere were monitored in Jinan, China. The average concentrations of phenol in PM2.5, TSP (total suspended particles), and gas-phase were 2.6 ± 0.4 ng/m^3^, 5.2 ± 0.8 ng/m^3^ and 11.3 ± 3.1 ng/m^3^ in summer, respectively [8]. Phenolic wastewater can be treated with activated carbon adsorption, chemical oxidation, electrochemical oxidation and biodegradation [9,10]. Biological methods are increasingly common for the treatment of industrial waste water. In recent decades, several phenol-degrading bacterial strains, such as *Pseudomonas * putida MTCC 1194 [11], *Rhodococcus* sp. [12], and *Candida tropicalis* sp. [13], have been isolated and characterized in different studies. Among these bacterial strains, *Rhodococcus* sp. can degrade multiple aromatic organic compounds such as quinoline, pyridine, catechol, and polychlorinated biphenyls (PCBs). *Rhodococcus* sp. strain AQ5NOL can tolerate up to 2000.0 mg/L with 1 mg/L of Zn^2+^ [14]. At the same time, Xu et al. demonstrated that *Rhodococcus* sp. can produce biosurfactants, which can be applied in eco-friendly methods to remove heavy metals, oil, and PCBs in industrial wastewater treatment [15]. The widespread distribution of *Rhodococcus* sp. combined with its capability to metabolize multiple pollutants suggests that *Rhodococcus* sp. may be a feasible alternative for treating phenolic waste waters

Metabolic kinetics studies can provide an in-depth analysis of the performance and mechanistic aspects during in situ bioremediation. To date, a wide range of kinetics models have been developed to simulate and predict the behavior of microbial degradation. Microbial biodegradation occurs in three phases. The first step involves a beginning phase at zero growth rate, and the second step occurs when the growth rate (*μ*) accelerates to the maximum over a certain period with a lag time. In the final phase, the decrease in degradation rate reaches zero [16]. During biodegradation, the maximum specific biomass growth rate (*μ*_max_), saturation constant for substrate (*K*_s_), maximum growth rate (*R_m_*), lag time (λ), and maximum yield coefficient (Y) constitute important parameters for understanding the biodegradability, affinity, and compatibility substrates to microorganisms. However, to the best of our knowledge, only a few studies have evaluated bacterial growth models and the degradation kinetics of phenol with *Rhodococcus* sp. in wastewater [17,18,19]. In this study, we isolated a strain which could degrade phenol at a higher concentration and provide a comprehensive evaluation of the degrading characteristic of *Rhodococcus* sp. SKC, via kinetic models for first time. The kinetic parameters of cell growth and degradation were quantified using different mathematical models including Haldane [20] and Gompertz kinetics [21,22]. The parameters, *μ*_max_ (the maximum specific biomass growth rate), *K*_s_ (saturation constant for substrate), *R_m_* (maximum growth rate), λ (lag time), and Y (maximum yield coefficient) were subsequently calculated and compared with other microorganisms, as these parameters play key roles in the effective design of biological methods to treat phenol contaminate (ground water, sanitary waste water).

## 2. Results and Discussion

### 2.1. Characterization of Rhodococcus sp. SKC

SKC was isolated from phenol-polluted soil and classified as Gram-positive bacteria. The 16S rRNA gene method was used for the phylogenetic analysis and the sequence of strain *Rhodococcus* sp. SKC was submitted to GenBank (MK342410). The similarity BLAST results indicate that the strain was closely related to *Rhodococcus* sp. with 99% sequence identity.

### 2.2. Biodegradation of Phenol as the Sole Carbon Substrate

**Figure 1:**
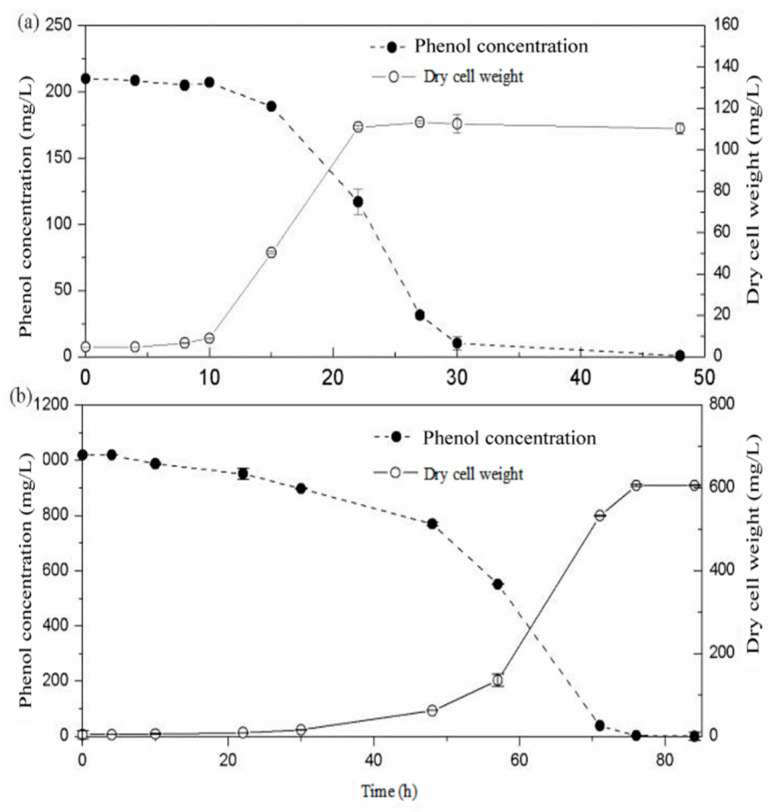
Degradation of phenol by *Rhodococcus* sp. SKC at initial concentrations of (**a**) 210.0 and (**b**) 1019.0 mg/L. Dry cell weight (DCW, mg/L) = 320.0 × OD_600_.

In order to understand the effect of concentration on *Rhodococcus* sp. SKC degradation, concentrations of 210.0 and 1019.0 mg/L were used to evaluate the removal ability of phenol. Figure 1a,b show the degradation profile of phenol at initial concentrations of 210.0 and 1019.0 mg/L at 30 °C. The results confirm that this strain can effectively remove phenol in aqueous solution and show a standard growth curve up to 1019.0 mg/L. During the biodegradation process, the influence of the lag phase was observed. The results show that the lag phase extended with a higher phenol concentration. When concentration reached 1500.0 mg/L (data not shown), there was no significant degradation or growth observed over 15 days. It is well known that phenol at high concentration is toxic to bacterium [23]. This observation clearly indicates that this strain exhibited a higher tolerance of phenol. However, inhibition was observed at high initial phenol concentrations due to the inhibitory nature of the target contaminant.

### 2.3. Effect of Temperature and Initial pH Value on Phenol Biodegradation

**Figure 2:**
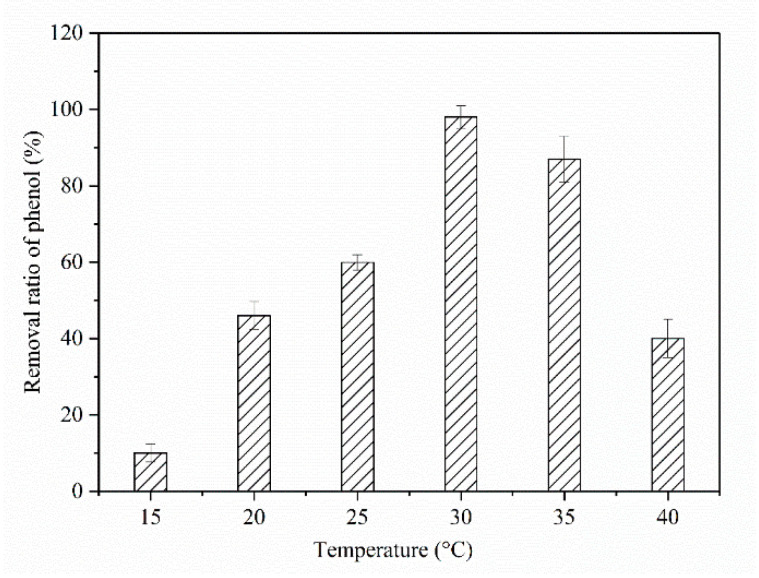
Effects of different temperatures on phenol degradation.

Considering that environmental parameters have a great effect on strain degradation, optimization was carried out for two parameters (i.e., pH and temperature). Optimization of these two parameters can lead to maximum degradation and a minimal lag phase [24,25,26]. Due to the fact that the enzymatic catalysis for the aromatic ring cleavage would be affected by temperature, a series of experiments were performed to establish the effect of temperature on phenol biodegradation rate and biomass, ranging from 15 to 40 °C (Figure 2). The results observe that phenol consumption rate was greatly influenced by temperature. The highest cell growth and phenol degradation rate were observed at 30 °C in lab-scale batch reactors.

**Figure 3:**
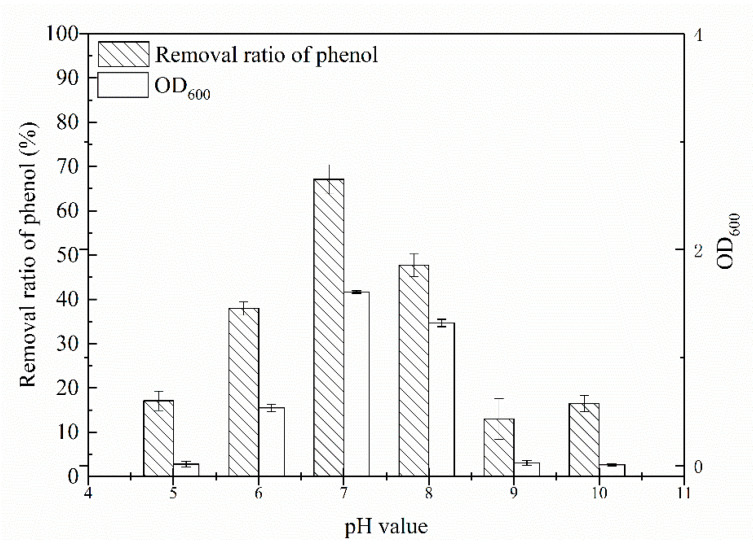
Effects of different initial pH values on removal ratio of phenol at 500.0 mg/L at 30 °C

Initial pH must influence the performance of the isolated bacteria during the treatment of phenol in waste water. Thus, the effect of pH (5.0 to 10.0) on the biodegradation of 500.0 mg/L phenol at 30 °C was studied in this work. Figure 3 shows that the optimum pH value for the strain to achieve maximum phenol removal ratio was at 7.0. *Rhodococcus* sp. SKC could degrade phenol at pH 5.0 (removal ratio of 17.08%), at an initial pH of 7.0, 67.10% of the phenol was removed after incubation for 42 h. When pH was at higher values, the removal percentage decreased to less than 15.0%. Cell density analysis indicated that *Rhodococcus* sp. SKC was able to utilize phenol as the sole carbon and energy source with the highest yield at a wide range of pH. To the best of our knowledge, phenolic waste water pH is usually at complicated condition [27]. Therefore, our results indicate that *Rhodococcus* sp. SKC could be used for wastewater treatment under similar conditions.

### 2.4. The Growth Kinetics

Phenol is toxic towards bacteria [23], and this toxicity could affect the integrity of the cytoplasmic membrane; this toxicity increases with phenol concentration. To understand the inhibition effect of phenol during biodegradation, a series of batch experiments were designed and conducted to determine the degradation and degradation rate kinetics. By applying these experimental data, the maximum phenol degradation rate and lag phase consumption data were obtained and simulated via the Gompertz Equation [21,22]:

(1) $$S^{}=S_{i}\times \left\{1-\exp\left\{-\exp\left[\frac{R_{m}}{s_{0}}e\left(\lambda -t\right)+1\right]\right\}\right\}$$

where *S* is the phenol concentration (mg/L), *S_i_* is the initial phenol concentration, *R_m_* is the maximum growth rate, λ is the lag phase time, and *t* is the time (h). All equations were run using Origin 8.0 software, and the best fit was calculated using a standard variance F-test.

**Figure 4:**
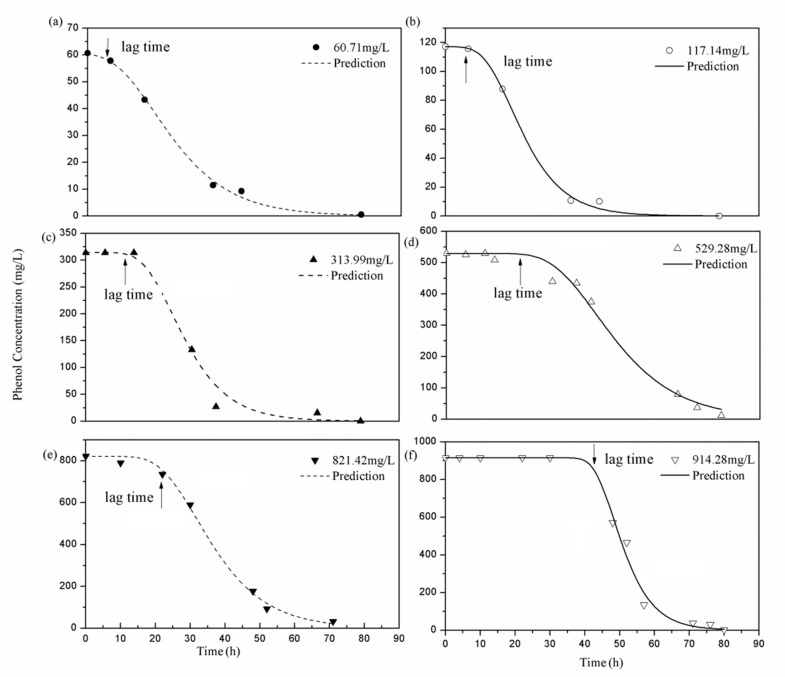
Fitting of *Rhodococcus* sp. SKC growth at different concentrations using the modified Gompertz model (**a**): 60.71 mg/L, (**b**): 117.14 mg/L, (**c**): 313.99 mg/L, (**d**): 529.28 mg/L, (**e**): 821.42 mg/L, (**f**): 914.28 mg/L. The arrow: the lag time (h).

Figure 4a–f reveal that the phenol degradation curves are well-described by the Gompertz model. In addition, the lag phase (λ) and the maximum phenol degradation rate for each initial phenol concentration were successfully fitted using this model (R^2^ > 0.98, RMSE < 0.01, *p* < 0.01). The *p*-value implies that the model corresponding to the degradation was significant. Table 1 shows the kinetic parameters of phenol degradation for each *S_i_*.

The lag time is the lag phase in the S-shaped curve of cell growth. The fitting results (Figure 4) show that the lag phase (λ) was extended with increasing the phenol concentration and the arrow shows this tendency. Relative to other degradation concentrations, 60.71 mg/L showed the shortest lag phase (4.56 h) (see Table 1). The lag time increased to 42.46 h when the initial phenol concentration increased to 914.28 mg/L. The maximum phenol growth rate (*R_m_*) increased as the initial phenol concentration increased from 3.08 to 55.79 mg/(L·h). This can be due to the toxicity of phenol on cells. The previous studies indicated that phenol can bring about changes in the cell membrane, and caused the increase in its permeability, leading the cell death [28]. Therefore, with higher concentrations of phenol, the cells take a longer time to adapt to the toxicity, and this results in the increased lag time. The results indicate that the strain was dose-dependent, and that the initial phenol concentration played a relevant role during biodegradation.

### 2.5. The Substrate-Inhibition Model

**Figure 5:**
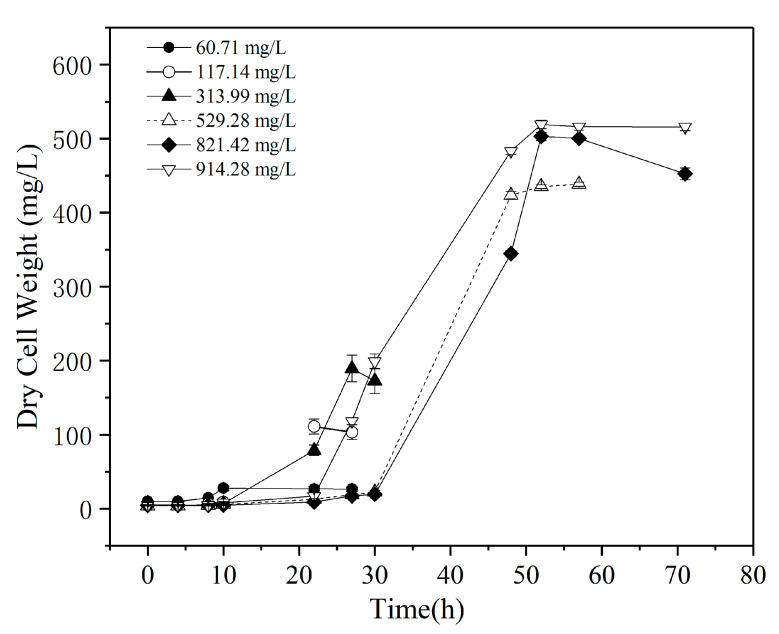
Increasing trend of biomass with ascending concentration of phenol from 60.0 to 914.28 mg/L.

To further describe the biodegradation process of phenol with *Rhodococcus* sp. SKC, a substrate-inhibition model that used the Haldane model was studied. The Monod equation is the most common kinetic model used to describe the bacterial biodegradation rate in a batch system [22]. However, the Monod equation can only describe ideal degraded conditions, and the Haldane model has been extensively accepted for describing and quantifying growth kinetics in response to substrate inhibition [29]. The biomass concentration (mg/L) of *Rhodococcus* sp. SKC for different initial phenol concentrations are shown in Figure 5, and the profiles can be modeled as:

(2) $$\frac{d_{X}}{d_{t}}=\mu X$$

(3) $$\frac{d_{S}}{d_{t}}=\frac{1}{Y_{X/t}}\frac{d_{X}}{d_{t}}$$

where *X* is the biomass concentration, *S* is the concentration of phenol, *t* is the time, *Y_x/__t_* is the yield coefficient, and *µ* is the specific growth rate.

The Haldane model can be used for higher phenol concentrations:

(4) $$\mu =\frac{\mu_{\max}\times S}{K_{S}+S+S^{2}/K_{i}}$$

where *µ*_max_ is the maximum specific growth rate, *K_s_* is the half saturation or affinity constant, and *K*_i_ is the inhibition constant.

The experimental data on a specific rate (*µ*) were obtained at various initial phenol concentrations (*S*) and used to estimate the kinetic parameters by employing the Haldane model.

**Figure 6:**
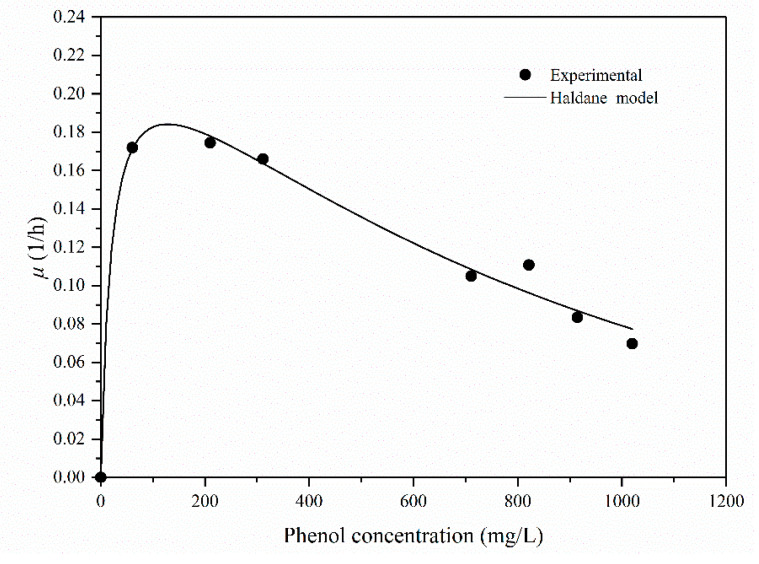
Phenol-specific growth rate profiles at the initial phenol concentration.

The phenol-specific growth rate profile at the initial phenol concentration is shown in Figure 6. An increasing biomass concentration was observed as the initial concentrations of phenol increased and were subsequently limited by higher phenol concentrations. The Haldane model parameters for the batch experiments were as follows: *μ*_max_ = 0.30 L/h, *K*_s_ = 36.40 mg/L and *K*_i_ = 418.79 mg/L. The correlation coefficient (R^2^) and RSME for this model were 0.9904 and 0.00925, respectively. In this figure (Figure 6), the specific growth rate increases at low phenol concentrations.

Table 2 summarizes the Haldane parameters for phenol biodegradation by different bacteria in previous studies. Comparison of the self-inhibition constants (*K*_i_) with those of other bacterial strains in the same range of initial phenol concentration shows that *Rhodococcus* sp. SKC has the highest value (418.79 mg/L). The *K*_i_ (the self-inhibition constants) value seen here shows that inhibition can be observed at 418.79 mg/L, indicating that *Rhodococcus* sp. SKC is less sensitive to higher initial phenol concentrations. This result also implies that *Rhodococcus* sp. SKC possessed higher tolerance and adaptation to a higher phenol concentration than other bacterial strains [11,14,30,31,32]. The *K_s_* (the half saturation) value of the model was 36.40 mg/L, which is in the middle range (0.13–99.03 mg/L) of that reported in the literature. This value indicates that this bacterium possessed a higher affinity toward the substrate than any other strain. The maximum specific growth rate (*μ*_max_) occurred at an initial phenol concentration of 123.45 mg/L, which is higher than those of *Pseudomonas putida* MTCC [11] and *Pseudomonas putida* LY1 [31] for the same phenol concentration range. These results show that *Rhodococcus* sp. SKC generally has a higher tolerance and adaptation to phenol than any other bacteria, thus demonstrating its applicability for the treatment of phenol-containing waste water over a wide range of concentrations of phenol.

### 2.6. Yield Factor

The biomass yield is an important parameter in assessing biodegradation performance. Experimental data on substrate degradation at different initial phenol concentrations were determined by plotting the biomass and the initial phenol concentration. The experimental values fit well (Equation (3)) with the R^2^ of the regression lines found between 0.90 and 0.98.

Table 3 shows the cell mass yield at different initial concentrations of phenol. The cell yield factor varies between 0.61 and 0.39 g/g as the phenol concentration varied from 60.71 to 914.20 mg/L. The maximum yield factor was observed at 313.90 mg/L (0.61 g/g), which is lower than the reported 0.83 g/g (93 mg/L) [30]. When inhibited, the cell mass yield increased with increasing initial phenol concentration, and the value remained nearly constant (Table 3). Notably, the cell mass yield was the sole indicator of the high metabolic efficiency of the bacteria. A comparison of these bacterial biodegradation parameters in other studies is given in Table 2, in which *Rhodococcus* sp. SKC could biodegrade phenol and still tolerate higher concentrations despite its biotoxicity, which can reduce the lag time and improve the bioremediation of phenol in industrial waste water [28].

## 3. Materials and Methods

### 3.1. Chemicals

High-purity phenol and inorganic salts of analytical reagent grade such as K_2_HPO_4_, KH_2_PO_4_, MgSO_4_·7H_2_O, NaCl, (NH_4_)_2_SO_4_ were obtained from Shengon Co., Ltd. (Shanghai, China). Peptone, beef extract, and agars were supplied by Sigma Co., Ltd. (St. Louis, MO, USA).

### 3.2. Isolation and Identification of Strain

Gram-positive *Rhodococcus* sp. SKC was isolated from phenol-polluted soil (China). *Rhodococcus* sp. SKC genomic DNA was extracted and amplified from 16S r RNA, and then sequenced based on the 16s genes. The universal primers were 27f (5′AGAGTTTGATCCTGGCT-CAG-3′) and 1492R (5′CGGCTACCTTGTTACGCTTC3′), and the reaction conditions consisted of an initial denaturation at 95 °C for 5 min, followed by 35 cycles at 94 °C for 1 min, 60 °C for 1 min, 72 °C for 1 min, and extension at 72 °C for 10 min. The polymerase chain reaction (PCR) products were sequenced by Shengon Co., Ltd. (Shanghai, China). The sequence was compared with existing BLAST data at the National Center for Biotechnology Information website (http://www.ncbi.nlm.nih.gov/).

### 3.3. Experimental

A pure strain of *Rhodococcus* sp. SKC was grown in mineral medium (MM) using phenol as the sole source of carbon at 30 °C under 120 rpm agitation. The MM used consisted of the following components: K_2_HPO_4_, 1.0 g; KH_2_PO_4_, 0.5 g, MgSO_4_·7H_2_O, 0.1 g; NaCl, 0.1 g; (NH_4_)_2_SO_4_, 1.0 g; and 10.0 mL of trace minerals per 1 L. The bacteria were collected by centrifuge (8000 rpm for 10 min at 25 °C). The harvested cells were washed twice and used as the inoculum for biodegradation testing.

Biodegradation experiments and inhibition test were carried out in 250 mL Erlenmeyer flasks with 99 mL of MM and 1 mL cell suspension (1%). The flask was incubated at 30 °C under 120 rpm agitation. Regarding optimistic estimates of pH, phenol concentration and temperature, different ranges were chosen. The initial phenol concentration varied from 50.0 to 1500.0 mg/L. The pH was studied in the pH range of 5.0, 6.0, 7.0, 8.0, 9.0 and 10.0. The temperature was in range of 15, 20, 25, 30, 35 and 40 °C, at 120 rpm. All samples were taken at scheduled intervals and analyzed cell density (OD_600_), concentration of phenol as the following methods. All tests were performed in triplicate, and the mean value is reported herein.

### 3.4. Analytical Methods

Phenol concentrations were quantified with high performance liquid chromatography (HPLC) (Shimadzu, Suzhou, China) using a Shimadzu LC-20AT system with a WAT 045905 symmetry C18 column (4.6 mm × 150 mm, 5 μm). Water samples were centrifuged at 13000 rpm for 3 min, and then syringe-filtered through 0.22 μm filters. The 20 μL samples were analyzed with HPLC using methanol: water (70:30 V/V) as the mobile phase at a flow rate of 1.0 mL/min; the UV detector was set at 280 nm with a retention time of 4.87 min. The biomass concentration was determined using a UV spectrophotometer (Shimadzu, Suzhou, China) at 600 nm by measuring the absorbance of the cell density (OD_600_) and then correlating the density to dry cell weight via the following equation: DCW (mg/L) = 320.0 × OD_600_. All experiments were performed in triplicate, and the results are reported as means with a standard deviation of ±3.0%.

## 4. Conclusions

The biological degradation of phenol with *Rhodococcus* sp. SKC in aqueous solution was evaluated in this study. The effect of pH and temperature, as well as the lag phase, maximum phenol degradation rate, maximum growth rate (*R_m_*), and maximum yield coefficient (Y) for each *S*_i_ (initial phenol concentration, mg/L), were evaluated using the Gompertz and Haldane models (R^2^ > 0.9904, RMSE < 0.00925). The values of these parameters at optimum conditions were *μ*_max_ = 0.30 (h^−1^), *K_s_* = 36.40 mg/L, and *K*_i_ = 418.79 mg/L. This illustrates that the inhibition concentration of phenol was 418.79 mg/L. When compared with other strains of bacteria, the biodegradation performance of *Rhodococcus* sp. SKC was higher and exhibited a higher tolerance to the inhibition of phenol. Collectively, these results show the potential application of *Rhodococcus* sp. SKC for the bioremediation of phenolic waste water.

## Figures and Tables

**Table 1:** Parameters and R^2^ using the Gompertz model on the substrate.

*S_i_* (mg/L)	*R_m_* [mg/(L·h)]	λ (h)	R^2^	*p*-Value
60.71	3.09	4.56	0.997	*p* < 0.001
117.14	8.67	6.62	0.997	*p* < 0.001
313.99	18.48	11.77	0.989	*p *< 0.01
529.28	21.00	21.80	0.988	*p* < 0.001
821.42	27.92	21.14	0.996	*p* < 0.001
914.28	55.79	42.46	0.991	*p* < 0.001

**Table 2:** Haldane model parameters of different bacteria reported previously.

Bacterial Strain	pH	Temperature (°C)	*S_i_* (mg/L)	*S*_max_ (mg/L)	*μ*_max_ (L/h)	*K_s_* (mg/L)	*K*_i_ (mg/L)	Yield (g/g)	References
*Sulfolobus solfataricus* 98/2	3.5	80	745.0	157.50	0.09	77.70	319.40	0.55	30
*Pseudomonas putida LY1*	7.2	25	800.0	50.00	0.27	24.40	127.40	0.77	31
*Pseudomonas putida* MTCC	7.0	30	1000.0	64.60	0.25	0.13	12.60	0.65	11
*Pseudomonas WUST-C1*	7.0	35	1600.0	-	2.50	48.70	100.60	-	32
*Rhodococcus* sp. *strain AQ5NOL 2.*	7.0	27	2000.0	-	0.11	99.03	354.0	-	14
*Rhodococcus* sp. SKC	7.0	30	1019.0	123.45	0.19	36.40	418.79	0.61	This work

**Table 3:** Cell mass yield at different initial concentrations.

*S_i_* (mg/L)	60.71	117.14	313.99	529.28	821.42	914.28
Y (mg biomass/mg substrate)	0.39	0.47	0.61	0.56	0.57	0.53
